# Storage-Induced Changes in Erythrocyte Membrane Proteins Promote Recognition by Autoantibodies

**DOI:** 10.1371/journal.pone.0042250

**Published:** 2012-08-03

**Authors:** Sip Dinkla, Věra M. J. Novotný, Irma Joosten, Giel J. C. G. M. Bosman

**Affiliations:** 1 Department of Laboratory Medicine - Laboratory of Medical Immunology, Radboud University Medical Centre, Nijmegen Institute for Infection Inflammation and Immunity, Nijmegen, The Netherlands; 2 Department of Hematology, Radboud University Medical Centre, Nijmegen, The Netherlands; 3 Department of Biochemistry, Radboud University Medical Centre, Nijmegen Centre for Molecular Life Sciences, Nijmegen, The Netherlands; Institut national de la santé et de la recherche médicale (INSERM), France

## Abstract

Physiological erythrocyte removal is associated with a selective increase in expression of neoantigens on erythrocytes and their vesicles, and subsequent autologous antibody binding and phagocytosis. Chronic erythrocyte transfusion often leads to immunization and the formation of alloantibodies and autoantibodies. We investigated whether erythrocyte storage leads to the increased expression of non-physiological antigens. Immunoprecipitations were performed with erythrocytes and vesicles from blood bank erythrocyte concentrates of increasing storage periods, using patient plasma containing erythrocyte autoantibodies. Immunoprecipitate composition was identified using proteomics. Patient plasma antibody binding increased with erythrocyte storage time, while the opposite was observed for healthy volunteer plasma, showing that pathology-associated antigenicity changes during erythrocyte storage. Several membrane proteins were identified as candidate antigens. The protein complexes that were precipitated by the patient antibodies in erythrocytes were different from the ones in the vesicles formed during erythrocyte storage, indicating that the storage-associated vesicles have a different immunization potential. Soluble immune mediators including complement factors were present in the patient plasma immunoprecipitates, but not in the allogeneic control immunoprecipitates. The results support the theory that disturbed erythrocyte aging during storage of erythrocyte concentrates contributes to transfusion-induced alloantibody and autoantibody formation.

## Introduction

Physiological, age-dependent removal of erythrocytes is an efficient and well-regulated process, consisting of controlled exposure of molecules that induce recognition of old erythrocytes by the immune system. This process includes senescent cell antigen formation on band 3, possibly in combination with phosphatidylserine (PS) exposure on the outer leaflet of the membrane and/or decreased CD47 expression, ultimately resulting in binding of autologous IgG and subsequent phagocytosis by macrophages of the reticulo-endothelial system. [1] During aging, the erythrocyte produces numerous vesicles, most of which expose PS, and that are enriched for IgG and age-related band 3 breakdown products. These vesicles are rapidly removed from the circulation, probably by the same mechanism that is responsible for erythrocyte removal. Vesiculation may constitute a protective mechanism to prevent untimely erythrocyte removal [2].

A clear picture of the molecular mechanisms involved in this age-dependent increase in removal signals is gradually emerging, and involves oxidative damage-induced, high-affinity binding of hemoglobin to band 3, activation of Ca^2+^-permeable channels, phosphorylation-controlled loss of metabolism and structure, and degradation and/or aggregation of band 3 fragments. However, the molecular details, triggers and cross-talk between these pathways are largely unknown [1].

Also, the erythrocyte contains a complex set of regulatory systems that may induce erythrocyte removal after physiological or pathological injury such as osmotic shock, oxidative stress and/or energy depletion. [3] Modulation of these pathways becomes progressively lost during storage, [4], [5] and this may result in accelerated aging and the removal of up to 30% of the transfused erythrocytes within 24 hours after transfusion. [6] Disruption of these systems may trigger aberrant expression of pathogenic epitopes on stored erythrocytes and their vesicles [7].

Frequent erythrocyte transfusions can lead to immunization and the formation of alloantibodies. This is especially problematic in the steadily increasing number of transfusion-dependent patients. Almost half of these patients acquire alloantibodies at some point in time, and in approximately 10% of the patients erythrocyte autoantibodies are detected. Part of the patients that produce these autoantibodies develop autoimmune hemolytic anemia (AIHA), which can be life-threatening [8].

We postulated that accelerated and/or altered erythrocyte aging during blood bank storage leads to the formation of non-physiological neoantigens that trigger the formation of autoantibodies. In order to test this hypothesis, we performed immunoprecipitations with erythrocytes and vesicles from blood bank concentrates of increasing storage periods, using plasma from patients containing erythrocyte autoantibodies. Subsequently, immunochemical and proteomic techniques were applied to identify the captured immune complexes. Our findings strengthen and deepen the view that disturbed erythrocyte aging during storage is related to transfusion-induced, anti-erythrocyte antibody formation.

## Materials and Methods

### Ethics

The study has been approved by the Committee on Research involving Human Subjects (CMO) of the Radboud University Medical Center (“Instituut Waarborging kwaliteit en veiligheid/Commissie Mensgebonden onderzoek regio- Arnhem-Nijmegen”) and in accordance with the declaration of Helsinki. Written informed consent was obtained from all blood donors participating in this study.

### Patients and Healthy Volunteers

Plasma samples from nine patients with a positive direct antiglobulin test (DAT) and confirmed erythrocyte autoantibodies were included in this study. Four patients were diagnosed with AIHA. One of these patients presented with AIHA after which a relapse acute myeloid leukemia was observed, while another was diagnosed with having both AIHA and anti-phospholipid syndrome. Two additional patients were diagnosed with immune thrombocytopenia and AIHA (Evans syndrome). Three patients with detectable erythrocyte autoantibodies without any clinical consequences was included as well (Table 1). The antibodies of one patient reacted with the erythrocyte Rhesus e-antigen on the patient’s autologous erythrocytes. In five patients anti-Wright^a^ (Wr^a^) antibodies were detected, and in one patient additional anti-C^w^ antibodies were present. Cold reactive autoantibodies were not detected in any of the samples. Allogeneic plasma from healthy volunteer blood donors was used as a control. All plasma samples used in this study had been stored at −20°C before use.

**Table 1:** Summary of patient information.

Patient	Clinical diagnosis	Blood group	DAT	IAT	Alloantibody	Autoantibody
1	AIHA (AML)	0 cc d ee	IgG, C3	1∶4	anti-Wr^a^	NS
2	Evans syndrome	0 cc d ee	IgG	1∶8	−	NS
3	AIHA (APLS)	AB CC D ee	IgG, C3	1∶1	anti-Wr^a^	NS
4	−	A CC D ee	IgG	1∶1	anti-Wr^a^, -C^w^	NS, anti-e
5	AIHA	A Cc D Ee	IgG, C3	1∶1	anti-Wr^a^	NS
6	AIHA	A Cc D ee	IgG	1∶4	anti-Wr^a^	NS
7	Evans syndrome	0 Cc D ee	IgG	1∶1	−	NS
8	−	0 Cc D ee	IgG	1∶1	−	NS
9	−	0 cc d ee	IgG, C3	1∶1	−	NS

DAT  =  direct antiglobulin test, IAT  =  indirect antiglobulin test (bovine) titer, AIHA  =  autoimmune hemolytic anemia, AML  =  acute myeloid leukemia, NS =  non-specific, APLS  =  antiphospholipid syndrome. All patients had a positive DAT and IAT.

### Isolation and Storage of Erythrocytes

An erythrocyte concentrate was obtained using standard blood bank procedures, from a single eligible donor who was AB0, Rhesus, Wr^a^ and C^w^ compatible for the detected antibodies in the patient plasmas. Whole blood (500 ml) was collected in a Composelect quadruple CPD-SAGM top-and-bottom bag system (Fresenius Kabi, Bad Homburg, Germany), containing 70 ml CPD as an anticoagulant. After cooling and centrifugation, erythrocytes were isolated using a Compomat G4 (Fresenius Kabi, Bad Homburg, Germany), after which 110 ml SAG-M was added to the erythrocytes. The erythrocyte suspension was leukocyte-depleted by in-line filtration, and subsequently stored at 2 to 6°C.

### Sampling of Erythrocyte Concentrates

During a storage period of 35 days, this erythrocyte concentrate was sampled at regular intervals. After sampling, erythrocytes were isolated by 10 min centrifugation at 1500 g. At the time of blood collection, an additional EDTA tube of whole blood was collected for isolation of plasma and fresh erythrocytes using a Ficoll gradient.

### Isolation of Erythrocyte Vesicles

Erythrocyte-derived vesicles were obtained from 35 day-old erythrocyte concentrates of two donors who were AB0, Rhesus and Wr^a^ compatible for the detected antibodies in the patient plasmas. The concentrates were centrifuged for 10 min at 1500 g to remove all cells. Membrane debris was then removed from the supernatant by centrifugation for 20 min at 1500 g. Vesicles were isolated by centrifuging 1.4 ml aliquots of supernatant for 20 min at 21,000 g. All but 25 µl of the supernatant was then removed, and the vesicle pellet was resuspended and stored at −80°C.

### Indirect Immunoprecipitation of Erythrocyte Autoantigens

Immunoprecipitation was performed using a modified version of the procedure described by Barker and colleagues. [9] Erythrocytes (1.5×10^9^) were washed three times using incomplete Ringer (IR) solution (32 mM HEPES, 125 mM NaCl, 5 mM glucose, 5 mM KCl, 1 mM MgSO_4_, pH 7.4), before incubation for 1 h at 37°C with 500 µl plasma diluted 1/1 in IR. The sensitized erythrocytes were then washed three times with IR, and lysed by adding lysis buffer (10 mM HEPES, 1 mM EDTA, 1 mM EGTA, 1 mM benzamidin, 5 µM leupeptin, pH 8.0). The erythrocyte membranes were pelleted by centrifugation at 21,000 g for 10 min and washed multiple times with lysis buffer to remove hemoglobin. The membranes were dissolved in 200 µl 1% TX-100 buffer (25 mM HEPES, 150 mM NaCl, 1 mM EDTA, 1 mM EGTA, 1 mM benzamidin, 5 µM leupeptin, pH 7.4) or RIPA buffer (1% NP-40, 1% deoxycholate, 0.1% SDS, 25 mM HEPES, 150 mM NaCl, 1 mM EDTA, 1 mM EGTA, 1 mM benzamidin, 5 µM leupeptin, pH 7.4) for 30 min on ice with regular vortexing. Unless mentioned otherwise, the TX100 buffer was applied. Insoluble cytoskeletal components were removed by 15 min centrifugation at 21,000 g. The protein content of the supernatant was determined using the Bradford assay. [10] Supernatant (250 µl) containing 0.35 mg protein was incubated with 50 µl protein G Dynabeads (Invitrogen, Carlsbad, USA) for 16 h at 4°C to capture immune complexes. The beads were washed three times with 1% TX-100 buffer or RIPA buffer prior to, and directly after the incubation with the supernatant. Then the captured proteins were dissociated in 15 µl Laemmli sample buffer (BioRad, Hercules, USA) containing 5% 2-mercaptoethanol for 30 min at 37°C. When non-denaturing conditions were applied, proteins were eluted using 50 mM glycine pH 2.8 for 5 min at room temperature, after which sample buffer was added (1/4 ratio) without 2-mercaptoethanol. Samples were stored at −80°C, and thawed on ice on the day of analysis. Immunoprecipitation of erythrocyte vesicles was performed using the same protocol without the lysis step, using a single vesicle aliquot per sample. Smaller (100 µl) incubation volumes were used for vesicle opsonization, membrane dissolution and immune complex capture. Plasma was depleted of vesicles by centrifugation for 60 min at 21,000 g before being used to opsonize erythrocyte vesicles. Subsequent erythrocyte vesicle isolation and washing was performed by centrifugation for 20 min at 21,000 g. Direct anti-band 3 immunoprecipitation in vesicles was performed using protein G Dynabeads, opsonized with a mouse monoclonal antibody that recognizes an N-terminal epitope of band 3 (BIII-136, Sigma-Aldrich, St. Louis, USA), diluted 1∶20 in 200 µl phosphate-buffered saline (PBS), pH 7.4. In the immunoprecipitation experiments we observed 25–30 and 50–60 kDa bands, representing the light and heavy chains of the bound antibodies, respectively.

### SDS-PAGE

SDS-PAGE was performed using TGX 4–15% gels in the Mini Protean 3 system (both BioRad, Hercules, USA). [11] Approximate molecular masses were calculated based on the Precision Plus Protein Standard (BioRad, Hercules, USA). Following SDS-PAGE (12.5 µl sample per lane), the gels were either used for immunoblotting, or developed using a silver stain. [12] Optical densities (OD) of the protein bands were determined using the GS 690 imaging densitometer (Bio-Rad, Hercules, USA) in combination with Molecular Analyst version 1.5 software. Total erythrocyte membrane fractions were loaded as positive controls and for normalization purposes.

### Erythrocyte Vesicle Membrane Protein Biotinylation

For immunoblotting, vesicle membrane proteins were biotinylated prior to IP. Vesicles were washed once with PBS pH 7.4, and labeled with 1 mM sulfo-NHS-biotin (Thermo Fisher, Waltham, USA) in PBS pH 8.0 for 30 min at 4°C. Residual sulfo-NHS-biotin was removed by two consecutive washing steps with PBS pH 7.4 containing 100 mM glycine.

### Immunoblotting

After SDS-PAGE, the proteins were transferred to PVDF membranes using the iBlot system (Invitrogen, Carlsbad, USA). The membranes were then blocked with Odyssey Blocking Buffer (OBB, LI-COR, Lincoln, USA), and incubated for 16 h at 4°C in OBB containing 0.1% Tween-20 and 1/1000 rabbit polyclonal antiserum against the membrane domain of human band 3 (K2N6B/PMB3, [13]). After three washing steps with PBS containing 0.1% Tween-20, the blots were incubated for 1 h at room temperature in OBB, 0.1% Tween-20, 0.01% SDS, 1/10,000 streptavidin-Alexa Fluor 680 (Invitrogen, Carlsbad, USA), and 1/10,000 goat anti-rabbit IgG-IRDye 800 (LI-COR, Lincoln, USA). This final incubation was followed by a single washing step using PBS containing 0.1% Tween-20, and three subsequent washes with PBS. Immunoblots were scanned using the Odyssey Infrared Imaging System (LI-COR, Lincoln, USA), and analyzed using Odyssey Software version 2.1.

### Proteomics

After one-dimensional gel electrophoresis and blue silver staining, [14] protein bands of interest were excised and submitted to in-slice tryptic digestion. In case of total protein identification, the sample was run briefly into the gel, after which the entire product was excised and digested. Peptide sequencing of tryptic digests was performed by nano-liquid chromatography tandem mass spectrometry using the LTQ-FT ICR (Thermo Fisher, Waltham, USA) mass spectrometer essentially as described previously. [15] Peptide and protein identifications were extracted with the Mascot search engine version 2.2, using the Reference Sequence (RefSeq) database at the National Center for Biotechnical Information (NCBI) with Homo sapiens taxonomy and added sequence-tags. Carbamidomethylation of cysteines (fixed), oxidation of methionine (variable) and acetylation of the N-terminus (variable) were the modifications allowed in the search. Protein identification validation was performed by an in-house developed script. [15] The software classifies protein identifications based on the number of uniquely identified peptide sequences, clusters proteins sharing the same set of peptides, and validates the proteins with the following criteria: proteins with a single peptide must have a peptide score of >49, proteins with multiple peptides must have a score of >29.

### Statistical Analysis

Differences between the patient and allogeneic control group were determined using a two-way ANOVA followed by a Bonferroni post test. Differences within a single group were determined using a one-way ANOVA followed by a Tukey post test. A confidence level of *p*<0.05 was considered to be significant.

## Results

### Altered Epitope Expression of Erythrocytes during Blood Bank Storage

Storage lesions, possibly resulting in accelerated aging, are responsible for the fast removal of a considerable portion of the erythrocytes after transfusion. Both non-physiological aging and enhanced removal are likely to contribute to the antibody responses against erythrocytes frequently observed in chronically transfused patients. In order to test the hypothesis that storage of erythrocytes under blood bank conditions leads to the formation of non-physiological neoantigens, a modified indirect immunoprecipitation was performed using plasma of six patients with erythrocyte autoantibodies (Table 1) or of healthy donors (see Materials and Methods), in combination with erythrocytes sampled at different time points from a stored erythrocyte unit. Immunoprecipitations were performed at regular intervals during blood bank storage.

**Figure 1:**
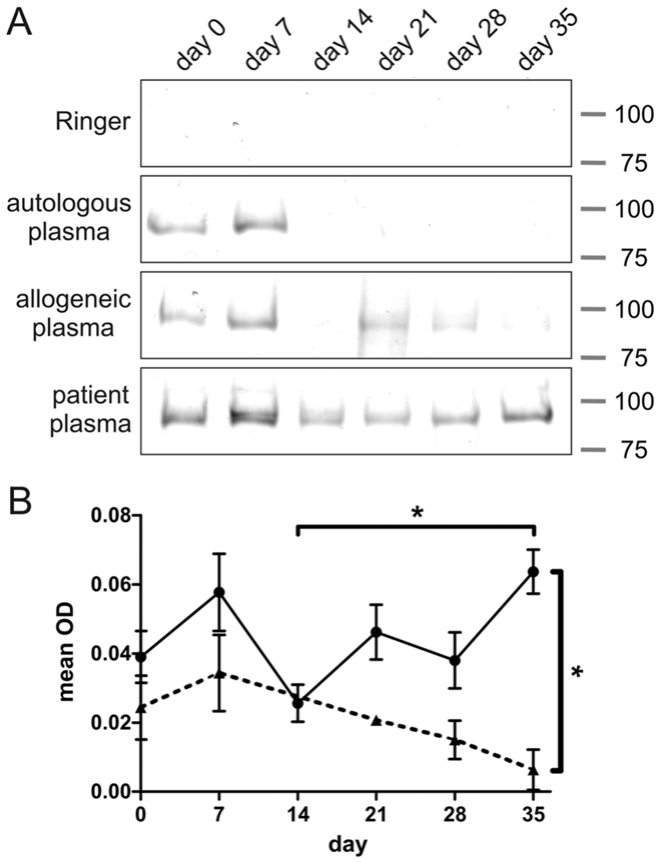
Erythrocyte autoantibody immunoprecipitation of erythrocytes sampled at regular time intervals during storage. Analysis was performed by SDS-PAGE, followed by silver staining. (A) Protein patterns of precipitates obtained using Ringer, autologous plasma, and a representative example from one out of three allogeneic plasmas, and one out of six autoantibody-containing plasmas (patient No. 2). For the allogeneic controls, day 14 is missing. (B) Mean optical density (OD) of patient (•, solid line, N = 6 patients) and allogeneic control plasma (▴, dotted line, N = 3 volunteers) precipitations. Numbers indicate approximate molecular weight (kDa). Error bars represent standard error, *p<0.05.

All plasmas tested precipitated proteins in the 90 to 100 kDa range (Figure 1). Immunoprecipitation using Ringer buffer instead of plasma did not result in any detectable protein precipitation (Figure 1A). Our data show that, although protein quantification of complex protein mixtures using silver staining can be problematic due to a limited dynamic range of the technique, [16] silver staining proved to work well with the highly purified immunoprecipitates we obtained (Figure 1A). Both the patient and the allogeneic control plasmas showed a decrease in signal after the first week of erythrocyte storage. In contrast to the control samples that revealed a further significant decrease in signal with time, the signals derived from patient plasma significantly increased again with storage time (Figure 1B). Immunoblot analysis of membrane fractions from erythrocytes of various storage periods using patient and allogeneic control plasma resulted in high background signals and/or non-specific binding, probably due to the denaturing conditions of SDS-PAGE and blotting (data not shown).

Freshly stored erythrocytes are recognized by naturally occurring antibodies. [1] At the later stages of storage, only autoantibodies present in the patient plasmas show enhanced binding to erythrocytes, suggesting a change in erythrocyte make-up upon storage that is only detectable with patient plasma. This change may be a trigger for pathological events. In order to determine the identity of the protein(s) involved, we proceeded to analyze the precipitated proteins by proteomics.

### Identity of the Precipitated Proteins

**Figure 2:**
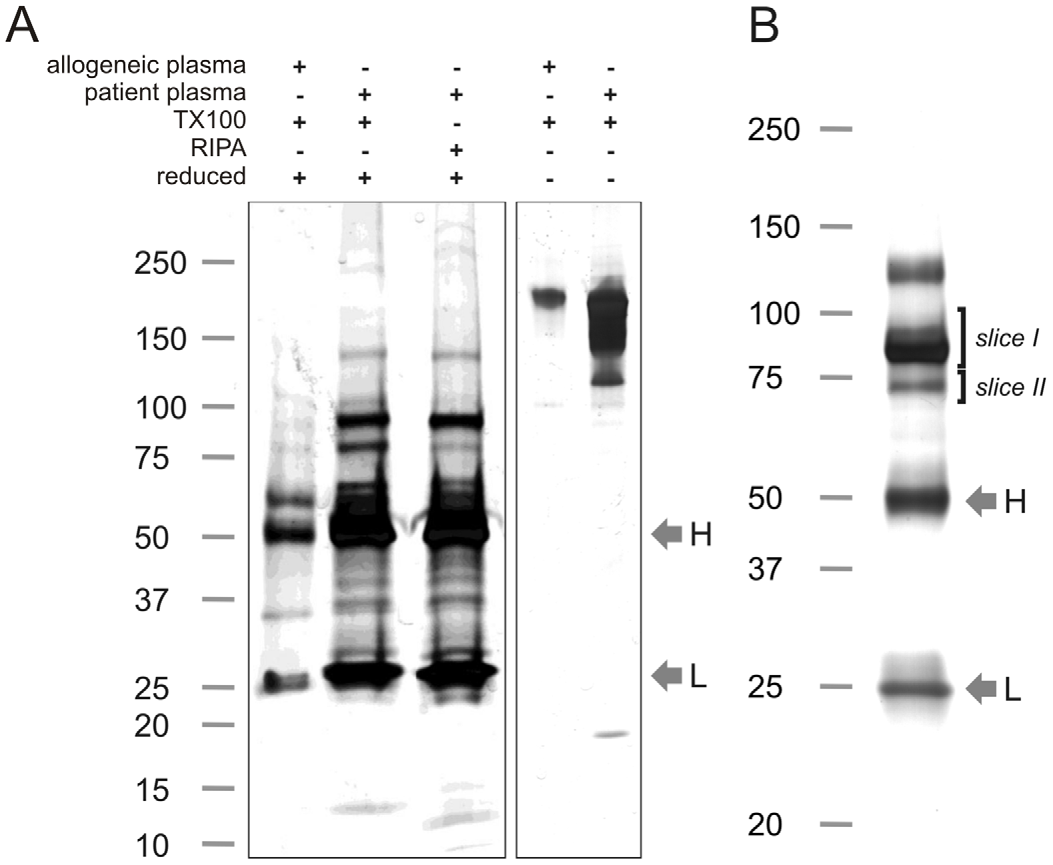
Erythrocyte autoantibody immunoprecipitation of stored erythrocytes. (A) Immunoprecipitation of 35 day old erythrocytes with erythrocyte autoantibody-containing patient plasma and allogeneic control plasma, using TX100 or RIPA extraction buffer and analyzed by SDS-PAGE under reducing or non-reducing conditions, followed by silver staining. A representative result (patient No. 2) from one out of three patient plasmas is shown. (B) Example of a silver stained gel of an immunoprecipitation of 35 day stored erythrocytes with plasma of patient No. 1. The same sample was used for Coomassie blue gel staining and subsequent proteomics analysis (Table 2). Gel slices which were excised for proteomic analyses are indicated as slices I and II (see also Table S1). Numbers indicate molecular weight (kDa). Heavy [H] and light [L] antibody chains are indicated by arrows.

In addition to the proteins in the 90 to 100 kDa range, immunoprecipitation of erythrocyte membrane fractions using patient plasmas revealed multiple other protein bands (Figure 2A). Differential extraction experiments showed that the proteins in the 90 to 100 kDa range were directly targeted by the patient plasma, instead of being co-precipitated as was the case for several other proteins, such as the 80 kDa protein band, which dissociated from the immune complex under the more stringent conditions of the RIPA buffer (Figure 2A). Furthermore, SDS-PAGE under non-reducing conditions indicated that the precipitated proteins mostly reside in one or more large complexes (Figure 2A). Heavy and light antibody chains (H and L in Figure 2A) are clearly visible due to the nature of the technique used.

For proteomics analysis, an immunoprecipitation using patient plasma (patients 1, 8 and 9) and allogeneic plasma (control) was performed in triplicate on erythrocytes stored for 35 days. A representative silver stained gel of one of these immunoprecipitations is depicted in Figure 2B. The total products of these immunoprecipitations were analyzed by mass spectrometry (Table 2). In addition, gel slices in the 80–100 and 70–80 kDa ranges (Figure 2B, slices I and II, respectively) were excised from the immunoprecipitation product of the erythrocytes incubated with the plasma of patient 1 and analyzed by proteomics.

**Table 2:** Summary of proteins identified by proteomics analyses of erythrocyte/vesicle immunoprecipitations using erythrocyte autoantibody-containing plasma of patients 1, 8 and 9, and allogeneic plasma (control).

	MW	Erythrocyte				Vesicle
Protein	(kDa)	Control	Patient 1	Patient 8	Patient 9	Patient 1
***Structural***						
Band 3	95		+	+		+
Band 4.1	66		+		+	+
Band 4.2	80			+	+	
Adducin	81		+		+	
Ankyrin	206					+
Actin	42		+	+	+	+
Spectrin	246					+
***Metabolism***						
GAPDH	36		+			+
Glucose transporter 1	54		+			+
Glutathione S-transferase	26		+			
Phosphofructokinase	85					
Type II PIP kinase	46					+
***Various***						
α globin	15		+			+
β globin	16		+	+	+	+
Annexin II	39					+
Carbonic anhydrase I	29		+			
Carbonic anhydrase II	29		+			+
HSP 70	70		+			
Stomatin	32		+			+
Thioredoxin	12		+			
Thrombospondin 1	129					+
Transglutaminase 3	77					
***Immunoglobulins***						
Ig heavy chain	50–60		+			+
Ig light chain	25–30		+			+
***Complement***						
CC 1	26			+	+	+
CC 3	187		+	+	+	+
CC 4	193	+	+	+	+	+
CC 5	188		+	+	+	+
CC 6	105				+	
CC 8	22				+	+
CC 9	63					+
Factor B	90			+	+	
***Complement inhibitors***						
C1 inhibitor				+	+	
Clusterin	58		+	+	+	+
Factor H	155			+	+	
Inter-α inhibitor	101–107		+	+	+	+
Vitronectin	54					+
***Lipoproteins***						
Apolipoprotein A	31		+	+	+	+
Apolipoprotein B	516	+	+	+	+	+
Apolipoprotein D	21					+
Apolipoprotein E Apolipoprotein H	36		+	+	+ +	+
Apolipoprotein L1	44					+

In summary, the proteomic analysis revealed that erythrocyte autoantibodies from the patient plasma precipitate multiple proteins. The samples consisted of membrane as well as cytosolic proteins, all of which have been described in previous proteomic inventories of the erythrocyte membrane (Table 2). [17] Furthermore they contained various plasma proteins, such as immunoglobulins, complement components, lipoproteins, and several immunoglobulins, and complement and coagulation-associated proteins. The protein compositionof the samples from the different patient samples revealed significant overlap, although less proteins were detected in the samples of patients 8 and 9. This could be due to the lower titer of these plasmas (Table 1). Proteomic analysis of the allogeneic plasma control precipitation did not reveal any erythrocyte-related proteins.

A complete list of all the proteins detected in the proteomics analysis is provided in Table S1.

Finally, we attempted to elucidate the nature of the most dominant antigens. The proteomic analysis of gel slice I (Figure 2B) identified several proteins, including band 3 as the only membrane protein detected (Table S1).

### Erythrocyte Autoantibodies Recognize Erythrocyte Vesicles Formed during Blood Banking

During their stay in the circulation, erythrocytes form vesicles that are rapidly removed once they appear in the bloodstream. Vesiculation also occurs during blood banking, especially during the later stages of storage. [18] Since erythrocyte vesiculation *in vivo* may constitute a mechanism for the removal of damaged membrane patches, and these vesicles are efficiently opsonized, [2], [15] we investigated whether vesicles formed during blood bank storage were also recognized by patient anti-erythrocyte antibodies.

**Figure 3:**
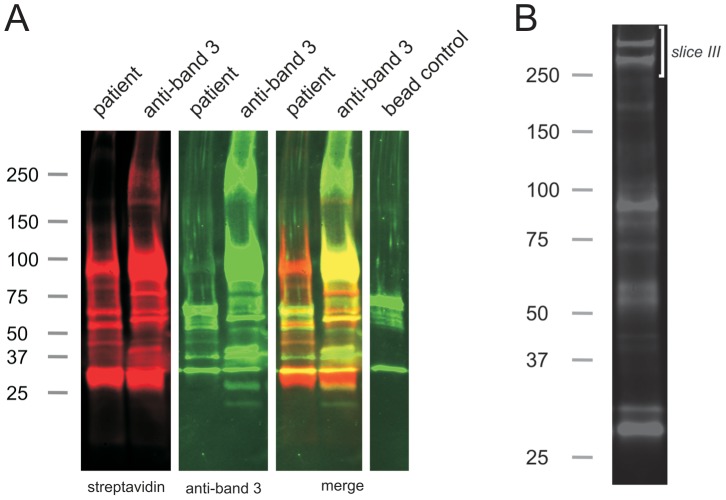
Erythrocyte autoantibody immunoprecipitation of biotinylated erythrocyte vesicles from a 35 day old transfusion unit. (A) Immunoprecipitation with either plasma from patient No. 2, or a monoclonal antibody against band 3 (see Materials and Methods). Analysis was performed by SDS-PAGE, followed by detection of biotinylated membrane proteins (red, streptavidin) and band 3 (green, polyclonal rabbit antibody). A protein G bead control was included. (B) Example immunoprecipitation of biotinylated erythrocyte vesicles from a 35 day old transfusion unit using plasma from patient No. 1. Analysis was performed by SDS-PAGE, followed by detection of biotinylated membrane proteins using fluorochrome conjugated streptavidin. The same sample was used for Coomassie blue gel staining and subsequent proteomics analysis (Table 2). The gel slice which was excised for proteomic analysis is indicated as slice III (see also Table S1). Numbers indicate approximate molecular weight (kDa). Blots were analyzed using the Odyssey Infrared Imaging System.

We performed immunoprecipitations using patient plasma and biotinylated erythrocyte vesicles isolated from 35 day old erythrocyte concentrates (see Materials and Methods). These precipitates were then visualized by immunoblotting for biotinylated membrane proteins.In stored erythrocyte vesicles, multiple proteins were targeted by the patient anti-erythrocyte antibodies, including the proteins in the 90 to 100 kDa range also observed in the erythrocyte precipitates (Figure 3A).

In order to identify the protein content of the targeted complex in these vesicles, an immunoprecipitation was performed in triplicate on vesicles obtained from a 35 day-old transfusion unit and analyzed by mass spectrometry (Table 2). An immunoblot of the immunoprecipitate is depicted in Figure 3B. In addition, we identified the proteins in a 240–320 kDa gel slice from the immunoprecipitation (Figure 3B, slice III and Table S1). A comparison of the erythrocyte to the vesicle precipitate shows a large overlap in their protein contents, including the presence of band 3. However, some clear differences were observed as well. Most notable are the absence of ankyrin and spectrin in the erythrocyte complex, and the absence of adducin in the vesicle complex (Table 2). In the vesicle immunoprecipitation, complement and lipoprotein peptides were much more abundant than in the erythrocyte immunoprecipitations (Table 2 and Table S1).

Since band 3 was found to be part of the vesicle-derived precipitates, immunoprecipitations using either patient plasma or a monoclonal anti-band 3 antibody and biotinylated erythrocyte vesicles were performed as well (Figure 3A). These precipitates were then visualized by immunoblotting for biotinylated membrane proteins and band 3. The latter immunoprecipitation revealed a biotinylated protein pattern that is different from that obtained with the patient plasma, indicating that different proteins were targeted (Figure 3A). Although band 3 staining of the patient sample immunoblot using a polyclonal anti-band 3 antiserum did not detect full-length band 3 (Figure 3A), several band 3 breakdown products that form during erythrocyte aging and storage were observed [1], [15].

## Discussion

During storage under blood bank conditions, erythrocytes undergo a number of functional and structural alterations, known as storage lesions. An accelerated and/or disturbed cellular aging process is likely to trigger aberrant expression of removal signals, thereby contributing to the removal of up to 30% of the erythrocytes within the first 24 hours after transfusion. This may contribute to the immunologic responses associated especially with chronic transfusions [1], [8].

Here we show that the main targets of the erythrocyte autoantibody-containing patient plasmas tested in this study are proteins in the 90 to 100 kDa range, which proteomic analysis revealed to include the membrane protein band 3. Band 3 is known to form three distinct complexes with other membrane and cytosolic proteins, and is the membrane anchorage site for the erythrocyte cytoskeleton. [19] Proteomic analysis showed that the band 3 binding partners adducin, ankyrin, band 4.1, band 4.2, GAPDH, hemoglobin and carbonic anhydrase were part of the precipitated immune complex, which suggests that band 3 complexes were indeed recognized. Although other candidate antigens cannot be completely ruled out, the observation that band 3 is the only membrane protein detected in the immunoprecipitates, makes it the most likely candidate antigen. Band 3 likely contains epitopes that trigger the harmful immune response leading to the formation of these erythrocyte autoantibodies. [20] This is underscored by the ability of a band 3 peptide to prime T cells for a band 3 response and accelerate the development of erythrocyte autoantibodies and anemia in a mouse model for AIHA [21].

Our data reveal that autologous and allogeneic plasma predominantly reacted with fresh and short stored erythrocytes, possibly by the binding of naturally occurring anti-band 3 antibodies. [1] In contrast, patient plasma autoantibody binding increased during erythrocyte storage, which suggests that non-physiological antigens become expressed during blood bank storage. One explanation for the enhanced autoantibody binding could be storage lesion-induced expression of erythrocyte aging-associated antigens [1], [4], [5].

Notably, previous work on erythrocyte autoantibodies, showed these antibodies to be specific for either band 3 or Rhesus protein. [9], [20] The apparent absence of Rhesus proteins in our analyses is probably due to the exclusion of patient plasma that showed (partial) specificity toward Rhesus antigens. The presence of alloantibodies against Wr^a^, an epitope located on band 3, [22] in five of the nine patients also hints at specificity for band 3 rather than Rhesus protein.

A remarkable observation was the precipitation of adaptor protein 2 (AP2) complex by the patient plasma (Table S1), as this protein is responsible for membrane attachment of, and membrane protein recruitment to clathrin-coated vesicles. [23] AP2 might be a remnant from the reticulocyte stage that binds to one or more proteins in the precipitated complex. Band 3 has been shown to interact with clathrin-coated vesicle machinery in kidney cells, which supports this possibility [24].

Although erythrocyte-derived vesicles formed during storage are known to be enriched in immunoglobulins, [15], [25] we here show that anti-erythrocyte autoantibodies readily recognized the vesicles as well. The vesicle precipitates contained spectrin and ankyrin, while the erythrocyte precipitates did not. The opposite was observed for adducin, which was present only in the erythrocyte precipitates. Ankyrin and adducin are known to reside in two functionally different complexes in the erythrocyte membrane, the ankyrin complex and the junctional complex. [19] An explanation for our observations may be the selective erythrocyte autoantibody binding of the band 3 - ankyrin complex in vesicles, compared to the selective targeting of the junctional complex in erythrocytes. The differences in protein composition between erythrocytes and their vesicles could also account for the absence of adducin in the vesicle precipitates. [2], [15], [18] The strikingly high complement content and apparent presence of autoantigens in the patient plasma vesicle precipitates indicate that these vesicles may be involved in clinically relevant immune responses.

The possibility of selective recognition of damaged or degraded band 3 in the vesicles was also investigated by comparing the patient plasma immunoprecipitation to that of a monoclonal anti-band 3 antibody. Patient erythrocyte autoantibodies appear to recognize a subset of the band 3 complexes in the vesicles, as there was only a partial overlap in the membrane proteins that were precipitated. This fits with the known selective binding of physiological autoantibodies to damaged band 3. [1], [4] The presence of damage-associated proteins (e.g. HSP70, 26S proteasome and transglutaminases) and band 3 degradation products in the complex precipitated using patient plasma supports this view (Table S1, and Figure 3, respectively) [26], [27].

Intriguingly, vesicle-associated spectrin was biotinylated using the membrane-impermeable sulfo-NHS-biotin (Table S1, and Figure 3C). One possible explanation is the diffusion of the sulfo-NHS-biotin into damaged vesicles via membrane pores. [25] Alternatively, the presence of inside-out oriented membrane vesicles could explain the presence of biotinylated spectrin. [28] The latter implies that, after erythrocyte transfusion, intracellular epitopes become accessible to the immune system, [29] a process generally known to be involved in the onset of autoimmune disorders [30].

The alternative erythrocyte autoantibody targeting in these vesicles, combined with the enhanced complement binding, support the notion that erythrocyte-derived vesicles might be important players in the inflammatory side-effects encountered during and after chronic erythrocyte transfusion. [8], [31] This is in line with the increasing amount of evidence showing that vesicles of different cellular origins are actively involved in inflammation.[32]–[34] Also, vesicle-containing supernatants from erythrocyte concentrates were found to have immune regulatory functions [35], [36].

Taken together, we have demonstrated a change in pathology-associated erythrocyte antigenicity during blood bank storage, which is accompanied with enhanced patient erythrocyte autoantibody binding. These changes probably include storage-related band 3 breakdown, as described in previous studies. [1], [4] The composition of the immune complex targeted in the vesicles was different from that of stored erythrocytes, implying that the vesicles might have a different capacity to modulate the immune system.

These findings corroborate the hypothesis that prolonged storage increases the transfusion-associated risks, [37] in particular the formation of anti-erythrocyte alloantibodies and autoantibodies by transfusion-dependent patients. We aim to elucidate the molecular identity of the involved epitopes, the mechanism(s) underlying these changes, and their pathophysiological implications.

## Supporting Information

Table S1 **Proteins identified by proteomics analyses of erythrocyte/vesicle immunoprecipitations using erythrocyte autoantibody-containing plasma of patients 1, 8 and 9, and allogeneic plasma (control).** Numbers represent the identified peptide sequences per protein. Either total products or gel slices containing proteins of a certain MW (kDa) were analyzed. Proteins in specific gel slices were also identified (slices I and II: Figure 2B, slice III: Figure 3B). Skin and trypsin contaminants were excluded from this overview. Immunoprecipitations and proteomics analyses were performed as mentioned in Materials and Methods.
(PDF)

## References

[1] Bosman GJ, Werre JM, Willekens FL, Novotny VM (2008) Erythrocyte ageing in vivo and in vitro: structural aspects and implications for transfusion. Transfus Med 18: 335–347. 19140816 10.1111/j.1365-3148.2008.00892.x

[2] Willekens FL, Werre JM, Groenen-Dopp YA, Roerdinkholder-Stoelwinder B, de Pauw B, et al (2008) Erythrocyte vesiculation: a self-protective mechanism? Br J Haematol 141: 549–556. 18419623 10.1111/j.1365-2141.2008.07055.x

[3] Lang KS, Lang PA, Bauer C, Duranton C, Wieder T, et al (2005) Mechanisms of suicidal erythrocyte death. Cell Physiol Biochem 15: 195–202. 15956782 10.1159/000086406

[4] Antonelou MH, Kriebardis AG, Stamoulis KE, Economou-Petersen E, Margaritis LH, et al (2010) Red blood cell aging markers during storage in citrate-phosphate-dextrose-saline-adenine-glucose-mannitol. Transfusion 50: 376–389. 19874562 10.1111/j.1537-2995.2009.02449.x

[5] Messana I, Ferroni L, Misiti F, Girelli G, Pupella S, et al (2000) Blood bank conditions and RBCs: the progressive loss of metabolic modulation. Transfusion 40: 353–360. 10738039 10.1046/j.1537-2995.2000.40030353.x

[6] Luten M, Roerdinkholder-Stoelwinder B, Schaap NP, de Grip WJ, Bos HJ, et al (2008) Survival of red blood cells after transfusion: a comparison between red cells concentrates of different storage periods. Transfusion 48: 1478–1485. 18482180 10.1111/j.1537-2995.2008.01734.x

[7] Fossati-Jimack L, zeredo da SS, Moll T, Kina T, Kuypers FA, et al (2002) Selective increase of autoimmune epitope expression on aged erythrocytes in mice: implications in anti-erythrocyte autoimmune responses. J Autoimmun 18: 17–25. 11869043 10.1006/jaut.2001.0563

[8] Young PP, Uzieblo A, Trulock E, Lublin DM, Goodnough LT (2004) Autoantibody formation after alloimmunization: are blood transfusions a risk factor for autoimmune hemolytic anemia? Transfusion 44: 67–72. 14692969 10.1046/j.0041-1132.2003.00589.x

[9] Barker RN, Casswell KM, Reid ME, Sokol RJ, Elson CJ (1992) Identification of autoantigens in autoimmune haemolytic anaemia by a non-radioisotope immunoprecipitation method. Br J Haematol 82: 126–132. 1419786 10.1111/j.1365-2141.1992.tb04604.x

[10] Bradford MM (1976) A rapid and sensitive method for the quantitation of microgram quantities of protein utilizing the principle of protein-dye binding. Anal Biochem 72: 248–254. 942051 10.1016/0003-2697(76)90527-3

[11] Laemmli UK (1970) Cleavage of structural proteins during the assembly of the head of bacteriophage T4. Nature 227: 680–685. 5432063 10.1038/227680a0

[12] Blum H, Beier H, Gross HJ (1987) Improved silver staining of plant proteins, RNA and DNA in polyacrylamide gels. Electrophoresis 8: 93–99.

[13] Bosman GJ, Visser FE, de Man AJ, Bartholomeus IG, de Grip WJ (1993) Erythrocyte membrane changes of individuals with Down’s syndrome in various stages of Alzheimer-type dementia. Neurobiol Aging 14: 223–228. 8321389 10.1016/0197-4580(93)90004-u

[14] Candiano G, Bruschi M, Musante L, Santucci L, Ghiggeri GM, et al (2004) Blue silver: a very sensitive colloidal Coomassie G-250 staining for proteome analysis. Electrophoresis 25: 1327–1333. 15174055 10.1002/elps.200305844

[15] Bosman GJ, Lasonder E, Luten M, Roerdinkholder-Stoelwinder B, Novotny VM, et al (2008) The proteome of red cell membranes and vesicles during storage in blood bank conditions. Transfusion 48: 827–835. 18346024 10.1111/j.1537-2995.2007.01630.x

[16] Grove H, Faergestad EM, Hollung K, Martens H (2009) Improved dynamic range of protein quantification in silver-stained gels by modelling gel images over time. Electrophoresis 30: 1856–1862. 10.1002/elps.200800568 [doi]. 10.1002/elps.200800568 19517441

[17] D’Alessandro A, Righetti PG, Zolla L (2010) The red blood cell proteome and interactome: an update. J Proteome Res 9: 144–163. 10.1021/pr900831f [doi]. 10.1021/pr900831f 19886704

[18] Salzer U, Zhu R, Luten M, Isobe H, Pastushenko V, et al (2008) Vesicles generated during storage of red cells are rich in the lipid raft marker stomatin. Transfusion 48: 451–462. 18067507 10.1111/j.1537-2995.2007.01549.x

[19] van den Akker E, Satchwell TJ, Williamson RC, Toye AM (2010) Band 3 multiprotein complexes in the red cell membrane; of mice and men. Blood Cells Mol Dis 45: 1–8. 20346715 10.1016/j.bcmd.2010.02.019

[20] Leddy JP, Falany JL, Kissel GE, Passador ST, Rosenfeld SI (1993) Erythrocyte membrane proteins reactive with human (warm-reacting) anti-red cell autoantibodies. J Clin Invest 91: 1672–1680. 8473510 10.1172/JCI116376 PMC288146

[21] Shen CR, Youssef AR, Devine A, Bowie L, Hall AM, et al (2003) Peptides containing a dominant T-cell epitope from red cell band 3 have in vivo immunomodulatory properties in NZB mice with autoimmune hemolytic anemia. Blood 102: 3800–3806. 10.1182/blood-2002-07-2125 [doi]. 10.1182/blood-2002-07-2125 12829598

[22] Bruce LJ, Ring SM, Anstee DJ, Reid ME, Wilkinson S, et al (1995) Changes in the blood group Wright antigens are associated with a mutation at amino acid 658 in human erythrocyte band 3: a site of interaction between band 3 and glycophorin A under certain conditions. Blood 85: 541–547. 7812009

[23] Lundmark R, Carlsson SR (2003) Sorting nexin 9 participates in clathrin-mediated endocytosis through interactions with the core components. J Biol Chem 278: 46772–46781. 10.1074/jbc.M307334200 [doi]. 10.1074/jbc.M307334200 12952949

[24] Sawasdee N, Junking M, Ngaojanlar P, Sukomon N, Ungsupravate D, et al (2010) Human kidney anion exchanger 1 interacts with adaptor-related protein complex 1 mu1A (AP-1 mu1A). Biochem Biophys Res Commun 401: 85–91. 20833140 10.1016/j.bbrc.2010.09.015

[25] Kriebardis AG, Antonelou MH, Stamoulis KE, Economou-Petersen E, Margaritis LH, et al (2008) RBC-derived vesicles during storage: ultrastructure, protein composition, oxidation, and signaling components. Transfusion 48: 1943–1953. 18564399 10.1111/j.1537-2995.2008.01794.x

[26] StolzA , Wolf DH (2010) Endoplasmic reticulum associated protein degradation: a chaperone assisted journey to hell. Biochim Biophys Acta 1803: 694–705. 10.1016/j.bbamcr.2010.02.005 [doi]. 10.1016/j.bbamcr.2010.02.005 20219571

[27] Facchiano A, Facchiano F (2009) Transglutaminases and their substrates in biology and human diseases: 50 years of growing. Amino Acids 36: 599–614. 10.1007/s00726-008-0124-8 [doi]. 10.1007/s00726-008-0124-8 18597041

[28] Willekens FL, Roerdinkholder-Stoelwinder B, Groenen-Dopp YA, Bos HJ, Bosman GJ, et al (2003) Hemoglobin loss from erythrocytes in vivo results from spleen-facilitated vesiculation. Blood 101: 747–751. 12393566 10.1182/blood-2002-02-0500

[29] Galletti J, Canones C, Morande P, Borge M, Oppezzo P, et al (2008) Chronic lymphocytic leukemia cells bind and present the erythrocyte protein band 3: possible role as initiators of autoimmune hemolytic anemia. J Immunol 181: 3674–3683. 18714043 10.4049/jimmunol.181.5.3674

[30] Racanelli V, Prete M, Musaraj G, Dammacco F, Perosa F (2011) Autoantibodies to intracellular antigens: Generation and pathogenetic role. Autoimmun Rev 10: 503–508. 10.1016/j.autrev.2011.03.001 [doi]. 10.1016/j.autrev.2011.03.001 21397735

[31] Hod EA, Zhang N, Sokol SA, Wojczyk BS, Francis RO, et al (2010) Transfusion of red blood cells after prolonged storage produces harmful effects that are mediated by iron and inflammation. Blood 115: 4284–4292. 20299509 10.1182/blood-2009-10-245001 PMC2879099

[32] Boilard E, Nigrovic PA, Larabee K, Watts GF, Coblyn JS, et al (2010) Platelets amplify inflammation in arthritis via collagen-dependent microparticle production. Science 327: 580–583. 20110505 10.1126/science.1181928 PMC2927861

[33] Couper KN, Barnes T, Hafalla JC, Combes V, Ryffel B, et al (2010) Parasite-derived plasma microparticles contribute significantly to malaria infection-induced inflammation through potent macrophage stimulation. PLoS Pathog 6: e1000744. 20126448 10.1371/journal.ppat.1000744 PMC2813278

[34] Mause SF, Weber C (2010) Microparticles: protagonists of a novel communication network for intercellular information exchange. Circ Res 107: 1047–1057. 10.1161/CIRCRESAHA.110.226456 [doi]. 10.1161/CIRCRESAHA.110.226456 21030722

[35] Vlaar AP, Hofstra JJ, Levi M, Kulik W, Nieuwland R, et al (2010) Supernatant of aged erythrocytes causes lung inflammation and coagulopathy in a “two-hit” in vivo syngeneic transfusion model. Anesthesiology 113: 92–103. 20508493 10.1097/ALN.0b013e3181de6f25

[36] Xiong Z, Cavaretta J, Qu L, Stolz DB, Triulzi D, et al (2011) Red blood cell microparticles show altered inflammatory chemokine binding and release ligand upon interaction with platelets. Transfusion 51: 610–621. 20738825 10.1111/j.1537-2995.2010.02861.x PMC3963470

[37] Koch CG, Li L, Sessler DI, Figueroa P, Hoeltge GA, et al (2008) Duration of red-cell storage and complications after cardiac surgery. N Engl J Med 358: 1229–1239. 10.1056/NEJMoa070403 [doi]. 10.1056/NEJMoa070403 18354101

