# Supplementary material for: Storage-Induced Changes in Erythrocyte Membrane Proteins Promote Recognition by Autoantibodies
Source: PLoS One. 2012 Aug 3;7(8):e42250. doi: 10.1371/journal.pone.0042250 (PMC3411782; doi:10.1371/journal.pone.0042250)
Supplement: Table S1 — Proteins identified by proteomics analyses of erythrocyte/vesicle immunoprecipitations using erythrocyte autoantibody-containing plasma of patients 1, 8 and 9, and allogeneic plasma (control). Numbers represent the identified peptide sequences per protein. Either total products or gel slices containing proteins of a certain MW (kDa) were analyzed. Proteins in specific gel slices were also identified (slices I and II: Figure 2B, slice III: Figure 3B). Skin and trypsin contaminants were excluded from this overview. Immunoprecipitations and proteomics analyses were performed as mentioned in Materials and Methods. (PDF) [file pone.0042250.s001.pdf]

Protein	*Erythrocyte*						*Vesicle*	
	Control	Patient 1	Patient 8	Patient 9	Slice I	Slice II	Patient 1	Slice III
**Actin**	0	1	3	3	2	0	1	0
**Adaptor-related protein complex 2, alpha 1 subunit**	0	0	0	0	6	1	0	0
**Adaptor-related protein complex 2, beta 1 subunit**	0	0	0	0	7	1	0	0
**Adducin 2**	0	5	0	2	1	0	0	0
Afamin	0	0	2	0	0	0	0	0
Aldehyde dehydrogenase 16 family, member A1	0	0	0	0	0	2	0	0
**Alpha globin**	0	5	0	0	0	1	2	3
Alpha-1-antichymotrypsin	0	0	7	7	0	0	0	0
Alpha-1-microglobulin/bikunin	0	0	0	2	0	0	0	0
Alpha-2-HS-glycoprotein	0	0	2	2	0	0	0	0
Alpha-2-macroglobulin	0	0	13	15	0	0	1	0
Angiotensinogen	1	0	2	3	0	0	0	0
**Ankyrin 1**	0	0	0	0	0	0	1	0
Annexin A2 isoform 2	0	0	0	0	0	0	1	0
Antithrombin III	0	0	5	5	0	0	0	0
**Apolipoprotein A**	0	2	7	6	0	0	1	0
**Apolipoprotein B**	2	35	22	33	0	0	63	2
**Apolipoprotein D**	0	0	0	0	0	0	1	0
**Apolipoprotein E**	0	3	0	4	0	0	8	0
**Apolipoprotein H**	0	0	3	2	0	0	0	0
**Apolipoprotein L1**	0	0	0	0	0	0	2	0
Arginase, type I	0	0	0	0	0	0	0	0
**Band 3 (solute carrier family 4, anion exchanger, member 1)**	0	6	3	0	5	3	2	0
**Beta globin**	0	9	3	1	4	3	6	4
Biliverdin reductase B (flavin reductase (NADPH))	0	1	0	0	0	0	0	0
**Carbonic anhydrase I**	0	2	0	0	0	1	0	0
**Carbonic anhydrase II**	0	1	0	0	0	0	1	0
Ceruloplasmin	2	0	8	9	0	0	2	0
Clusterin	0	4	3	3	0	0	4	0
**Complement C1s subcomponent**	0	0	2	2	0	0	0	0
**Complement C6**	0	0	0	1	0	0	0	0
**Complement component 1, q subcomponent, B chain**	0	0	1	2	0	0	2	0
**Complement component 1, q subcomponent, C chain**	0	0	2	2	0	0	3	2
**Complement component 3**	0	12	3	2	0	2	20	0
**Complement component 4**	5	1	16	17	0	0	4	0
**Complement component 5**	0	4	3	4	0	0	24	0
**Complement component 8, gamma polypeptide**	0	0	0	1	0	0	1	0
**Complement component 9**	0	0	2	2	0	0	6	0
**Complement factor B**	0	0	3	2	0	0	0	0
Complement factor H isoform a	0	0	5	4	0	0	0	0
Corticotropin releasing hormone	0	3	0	0	2	1	2	2
Cyclin M2	0	1	0	0	2	2	2	3
**Erythrocyte membrane protein band 4.1**	0	1	0	4	1	11	5	0
**Erythrocyte membrane protein band 4.2**	0	0	1	1	0	0	0	0
Eukaryotic translation initiation factor 2C, 2	0	0	0	0	7	4	0	0
Eukaryotic translation initiation factor 4A	0	0	0	0	0	1	0	0
Fibrinogen alpha chain	0	0	2	4	0	0	0	0
Fibrinogen beta chain	0	0	4	2	0	0	0	0
Fibrinogen gamma chain	0	0	5	4	0	0	0	0
Fibronectin 1	0	0	1	1	0	0	2	3
Glutathione S-transferase	0	1	0	0	0	0	0	1
**Glyceraldehyde-3-phosphate dehydrogenase**	0	1	0	0	1	0	1	0
**Heat shock 70kDa protein**	0	1	0	0	0	0	0	0
Hemopexin	0	0	6	4	0	0	0	0
Heparin cofactor II	0	0	2	2	0	0	3	0
Histidine-rich glycoprotein	0	0	3	2	0	0	2	0
**Ig heavy chain**	0	3	0	0	3	3	2	0
**Ig heavy chain**	0	3	0	0	3	2	1	0
**Ig heavy chain**	0	1	0	0	2	2	0	0
**Ig heavy chain**	0	1	0	0	1	1	1	0
**Ig heavy chain**	0	0	0	0	1	1	0	0
**Ig light chain**	0	1	0	0	0	1	1	0
**Ig light chain**	0	1	0	0	0	1	1	0
**Ig light chain**	0	1	0	0	1	1	1	0
**Ig light chain**	0	1	0	0	1	1	1	0
Insulin-like growth factor-binding protein complex acid labile subunit	0	0	0	2	0	0	0	0
Inter-alpha (globulin) inhibitor H1	0	0	5	4	0	0	4	2
Inter-alpha (globulin) inhibitor H2	0	2	4	3	0	0	5	1
Inter-alpha (globulin) inhibitor H4	0	1	7	6	0	0	4	0
Kininogen-1 isoform 2	0	0	6	4	0	0	0	0
Leucine-rich alpha-2-glycoprotein	0	0	0	3	0	0	0	0
Lipocalin 1	0	0	0	0	0	0	1	0
Lipopolysaccharide-binding protein	0	0	0	0	0	0	2	0
Liver phosphofructokinase	0	0	0	0	0	5	0	0
Lysozyme	0	0	2	0	0	0	1	0
Neuroblastoma RAS viral (v-ras) oncogene homolog	0	0	0	0	0	0	1	0
Peptidylprolyl isomerase B	0	0	0	0	0	0	0	0
Phosphatidylinositol-5-phosphate 4-kinase, type II, alpha	0	0	0	0	0	0	1	0
Plasma protease C1 inhibitor	0	0	3	2	0	0	0	0
Plasminogen	0	0	2	0	0	0	0	0
Prolactin-induced protein	0	0	0	0	0	0	1	0
**Proteasome 26S non-ATPase subunit 2**	0	0	0	0	2	0	0	0
Prothrombin	0	0	5	3	0	0	0	0
Serine (or cysteine) proteinase inhibitor, clade A, member 10	0	0	0	0	0	0	1	0

Serine (or cysteine) proteinase inhibitor, clade A, member 4	0	0	0	0	0	0	1	0
Serine (or cysteine) proteinase inhibitor, clade B, member 12	0	0	0	0	0	0	0	0
Serine (or cysteine) proteinase inhibitor, clade B, member 4	0	0	0	0	1	0	0	0
Serotransferrin	0	0	4	3	0	0	0	0
Serpin peptidase inhibitor, clade A, member 3	0	0	0	0	0	0	1	0
Serum albumin	0	0	4	0	0	0	0	0
Serum amyloid P-component	0	0	2	3	0	0	0	0
Solute carrier family 2 (facilitated glucose transporter), member 1	0	1	0	0	1	2	1	0
**Sorting nexin 9**	0	0	0	0	0	1	0	0
**Spectrin beta**	0	0	0	0	0	0	1	2
Stomatin isoform a	0	2	0	0	0	0	10	0
Thioredoxin	0	1	0	0	0	0	0	0
Thrombospondin 1	0	0	0	0	0	0	2	0
Thyroxine-binding globulin	0	0	2	1	0	0	0	0
**Transglutaminase 2 isoform a**	0	0	0	0	0	2	0	0
**Transglutaminase 3**	0	0	0	0	0	0	0	0
Transmembrane protein 24	0	1	0	0	1	0	0	0
Ubiquitin and ribosomal protein	0	1	0	0	1	1	1	1
Urocortin	0	0	0	0	0	0	1	2
Vitronectin	0	0	0	0	0	0	2	0
Von Willebrand factor	0	0	0	0	0	0	4	0

Table S1. Proteins identified by proteomics analyses of erythrocyte/vesicle immunoprecipitations using erythrocyte autoantibody-containing plasma of patients 1, 8 and 9, and allogeneic plasma (control). Numbers represent the identified peptide sequences per protein. Either total products or gel slices containing proteins of a certain MW (kDa) were analyzed. Proteins in specific gel slices were also identified (slices I and II: Figure 2B, slice III: Figure 3B). Skin and trypsin contaminants were excluded from this overview. Immunoprecipitations and proteomics analyses were performed as mentioned in Materials and Methods.
